# PoSSuM v.2.0: data update and a new function for investigating ligand analogs and target proteins of small-molecule drugs

**DOI:** 10.1093/nar/gku1144

**Published:** 2014-11-17

**Authors:** Jun-ichi Ito, Kazuyoshi Ikeda, Kazunori Yamada, Kenji Mizuguchi, Kentaro Tomii

**Affiliations:** 1 Laboratory of Bioinformatics, National Institute of Biomedical Innovation (NIBIO), 7-6-8 Saito-Asagi, Ibaraki, Osaka 567-0085, Japan; 2 Computational Biology Research Center (CBRC), National Institute of Advanced Industrial Science and Technology (AIST), 2-4-7 Aomi, Koto-ku, Tokyo 135-0064, Japan; 3 Drug Discovery Informatics Group, System Solution Division, Level Five Co. Ltd., Shiodome Shibarikyu Bldg., 1-2-3 Kaigan, Minato-ku, Tokyo 105-0022, Japan

## Abstract

PoSSuM (http://possum.cbrc.jp/PoSSuM/) is a database for detecting similar small-molecule binding sites on proteins. Since its initial release in 2011, PoSSuM has grown to provide information related to 49 million pairs of similar binding sites discovered among 5.5 million known and putative binding sites. This enlargement of the database is expected to enhance opportunities for biological and pharmaceutical applications, such as predictions of new functions and drug discovery. In this release, we have provided a new service named PoSSuM drug search (PoSSuMds) at http://possum.cbrc.jp/PoSSuM/drug_search/, in which we selected 194 approved drug compounds retrieved from ChEMBL, and detected their known binding pockets and pockets that are similar to them. Users can access and download all of the search results via a new web interface, which is useful for finding ligand analogs as well as potential target proteins. Furthermore, PoSSuMds enables users to explore the binding pocket universe within PoSSuM. Additionally, we have improved the web interface with new functions, including sortable tables and a viewer for visualizing and downloading superimposed pockets.

## INTRODUCTION

The number of released protein entries in the Protein Data Bank (PDB) (1) has reached 100 000. To elucidate protein functions from the abundant structural data, an efficient approach must be used to examine ligand-binding sites specifically because proteins exhibit their functions through interaction with other molecules. Of particular interest are small-molecule binding pockets, which are crucial in structure-based drug discovery. To retrieve similar binding pockets of biological relevance, many methods have been proposed in the last few decades (2–9). However, those methods are applicable only to a limited subset of protein binding pockets mainly because of the time complexity. We developed an extremely fast and efficient method for finding similarities between vast numbers of ligand-binding pockets in our previous study (10,11) and then applied it to all-against-all similarity searches against 1.8 million known and putative small molecule-binding pockets throughout the PDB. Eventually, we discovered 14 million pairs of similar binding sites. All enumerated similar pairs, along with biological annotations, were compiled into the PoSSuM database (http://possum.cbrc.jp/PoSSuM/) (12).

Since its initial release (2011), PoSSuM has grown to include more than 49 million pairs of similar binding sites identified among about 5.5 million binding sites. This database expansion is expected to enhance opportunities for biological and pharmaceutical applications such as the prediction of new protein functions. The binding pockets were assigned with UniProt (13) identifiers, Enzyme Commission (EC) numbers (14), Gene Ontology (15) terms, and domain annotations from CATH (16), SCOP (17) and SCOPe (18), and compiled into a relational database.

A PoSSuM similarity search starts by choosing a known ligand-binding pocket (SearchK) or a whole protein structure (SearchP) as a query. However, it is also useful to search for similar binding pockets that bind a specific type of ligand, such as a small-molecule drug. The orally available small-molecule drugs are regarded as pharmaceutically important chemicals because of their safety and simple applicability. Therefore, finding an unexpected similarity between a known drug target and an unknown one might provide a hint for consideration of an ‘off-target effect’ of the corresponding drug. Another important consideration is that these small-molecule drugs have a wealth of drug discovery data: bioactivity data, medicinal chemistry data and target information. Therefore, it is possible to verify the similarity between proteins that have similar binding pockets even in the absence of protein-ligand complex data. In this study, we performed a survey, in which we focused on 194 bioactive small-molecule drugs with ligand-protein complex structures available in the PDB, and compared their binding pockets against the entire PDB by using a PoSSuM similarity search. The results were compiled into a new web interface, PoSSuMds, which is expected to be useful for investigating analogs of drugs and also for fishing for potential targets.

In addition to this update and new developments described above, we have introduced new features including redundancy removal on the result page, sortable tables and the improved molecular viewer, as well as a downloading function of the coordinates of superimposed pockets.

## DATA GROWTH TO OVER 4.7 MILLION BINDING SITES

As of September 2014, the PoSSuM data source contains 49 million pairs of similar binding sites detected between 4.7 million binding pockets (Table 1). Since its initial release in November 2011, the number of binding pockets has increased by approximately three times. This data expansion is attributable not only to our annual updates on the release of new PDB entries, but also to the results from the removal of the use of a representative subset. Previously, putative binding pockets were detected from non-redundant protein structures (defined with a cutoff of 95% sequence identity) by using Ghecom, a novel pocket prediction method (19). In the new release, all PDB protein structures, except for structures with a resolution of less than 4.0 Å or containing more than 3000 residues, are used to generate putative pockets. This extension not only increases the opportunity to obtain hits, but also provides a more convenient service in ‘SearchP’, in which a PDB ID is specified as a query.

**Table 1.** Data growth of PoSSuM since the initial release

	Initial release	Up-to-date
	Nov. 2011	Sept. 2014
PDB version	Jan. 2011	May 2014
No. of known ligand-binding sites	241 486	300 122
(No. of PDB entries)	(47 562)	(65 450)
No. of putative binding sites	1 588 329	5 213 569
(No. of PDB entries)	(29 779)	(88 290)
Pairs of similar binding sites	14 556 057	49 078 742

Given a query of a binding pocket, PoSSuM enumerates all similar pockets irrespective of the sequence redundancy. However, users can filter out the redundancy by setting a UniProt/UniRef50 filter. If multiple similar pockets with the same UniProt ID and HET code are identified, then the pairs with the longest aligned length can be displayed in the case of a UniProt filter. In the case of a UniRef50 filter, the UniProt ID was substituted with the UniRef50 ID. In this event, the longest matched pairs were displayed.

## PoSSuM DRUG SEARCH (PoSSuMds)

**Figure 1.**
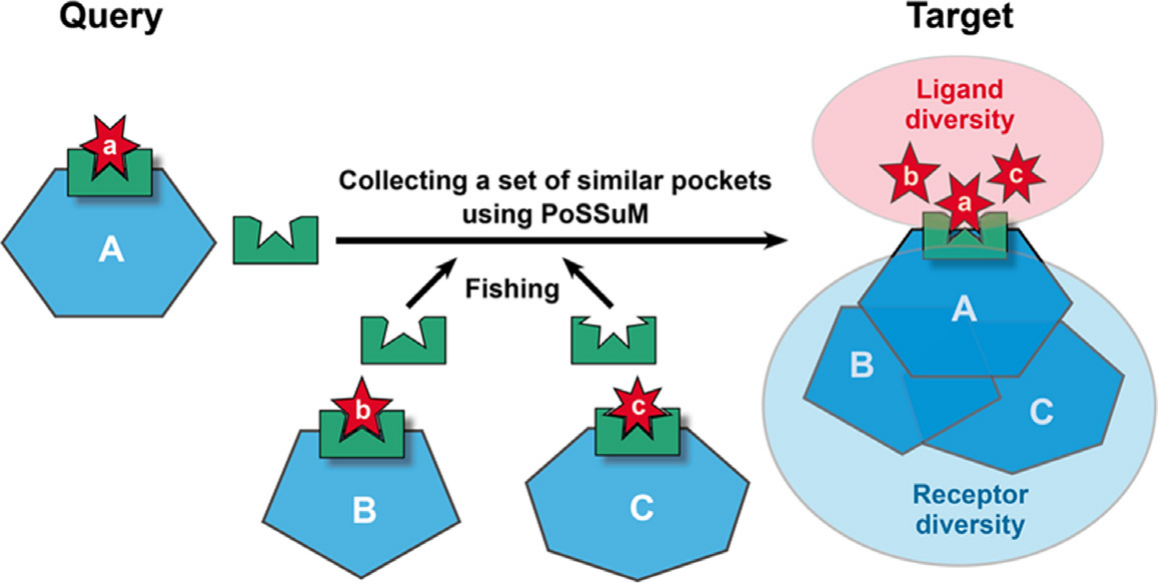
Schematic view of the PoSSuMds approach for screening ligand analogs and their receptor proteins. Ligands, binding pockets and receptors are shown in red, green and cyan, respectively.

For any given drug compound, a challenging task in structural bioinformatics is to find related (and biologically relevant) compounds and target proteins. Our approach is to collect a set of binding sites similar to the pockets to which the query ligand is known to bind. This type of approach is useful for retrieving various ligand analogs, such as natural ligands, inhibitors and metabolites, and also for obtaining potential target proteins (Figure 1).

### Small-molecule drug data set and results of a similarity search

Small-molecule drug compounds and their chemical properties were retrieved from the ChEMBL database (20). From ChEMBL release 19, we selected all of the drug compounds that fulfilled the following criteria:

- Approved drug (molecule_dictionary.max_phase = 4)
- Used as a therapeutic (molecule_dictionary. therapeutic_flag > 0)
- Oral drug (molecule_dictionary. oral > 0)
- Non-prodrug (molecule_dictionary.prodrug = 0)

Of these, 211 unique drug compounds were identified as having at least one protein-ligand complex structure in the PDB by matching the standard InChIKey against the Ligand Expo database (21). The PoSSuM source data included 194 such drug ligands (HET codes), which have at least one binding pocket showing similarities to different pockets. In all, 2595 binding pockets for the 194 ligands were used as queries in this study (Table 2). Query pockets were then searched against all of the non-self pockets by using PoSSuM. Subsequently, 530 898 similarities were detected between 26 509 unique pockets, which were occupied by 5312 unique ligands (HET codes) and derived from 12 220 unique protein structures (PDB IDs). Finally, all of the results were compiled into individual web pages based on the name of the query drug (HET codes 1–194).

**Table 2.** Statistics of small-molecule drug search results

	Query	Target
Pocket similarities	530 898	
Binding pockets	2595	26 509
Ligands (HET codes)	194	5312
PDB entries (PDB IDs)	1289	12 220
UniProt entries	452	2453
UniRef50 entries	384	1969
EC numbers	165	521
CATH homologous super families	108	384
CATH topologies	80	255
SCOPe super families	97	376
SCOPe folds	86	287

### Diversity of identified ligands and receptors

To describe the diversity of the retrieved ligands, we compared the ligand structures by using two types of molecular fingerprints: MACCS (166 bits) and Babel FP2 (1024 bits), in which ligand compounds were encoded into fingerprints by OpenBabel (http://openbabel.sourceforge.net/). Similarities between them were then measured by using the Jaccard Index (JI) metric. To represent relationships between ligands graphically, two ligands were connected with an edge if their JI (MACCS) was ≥ 0.568 or JI (FP2) was ≥ 0.318, where the statistical significance (*P*-value < 0.05) of each JI threshold was estimated based on 1 million randomly generated pairs of ligands among all of the HET compounds used for this study (Supplementary Figure S1). To investigate the diversity of retrieved receptors, we compared functional and structural classifications, which included EC numbers, CATH homologous super families and SCOPe super families, between query proteins and target proteins identified by pocket similarity searches.

To provide users with better and dynamic graphics, all figures on the result page were generated with D3.js (https://github.com/mbostock/d3), a Java Script library.

## WEB INTERFACE OF PoSSuMds

**Figure 2.**
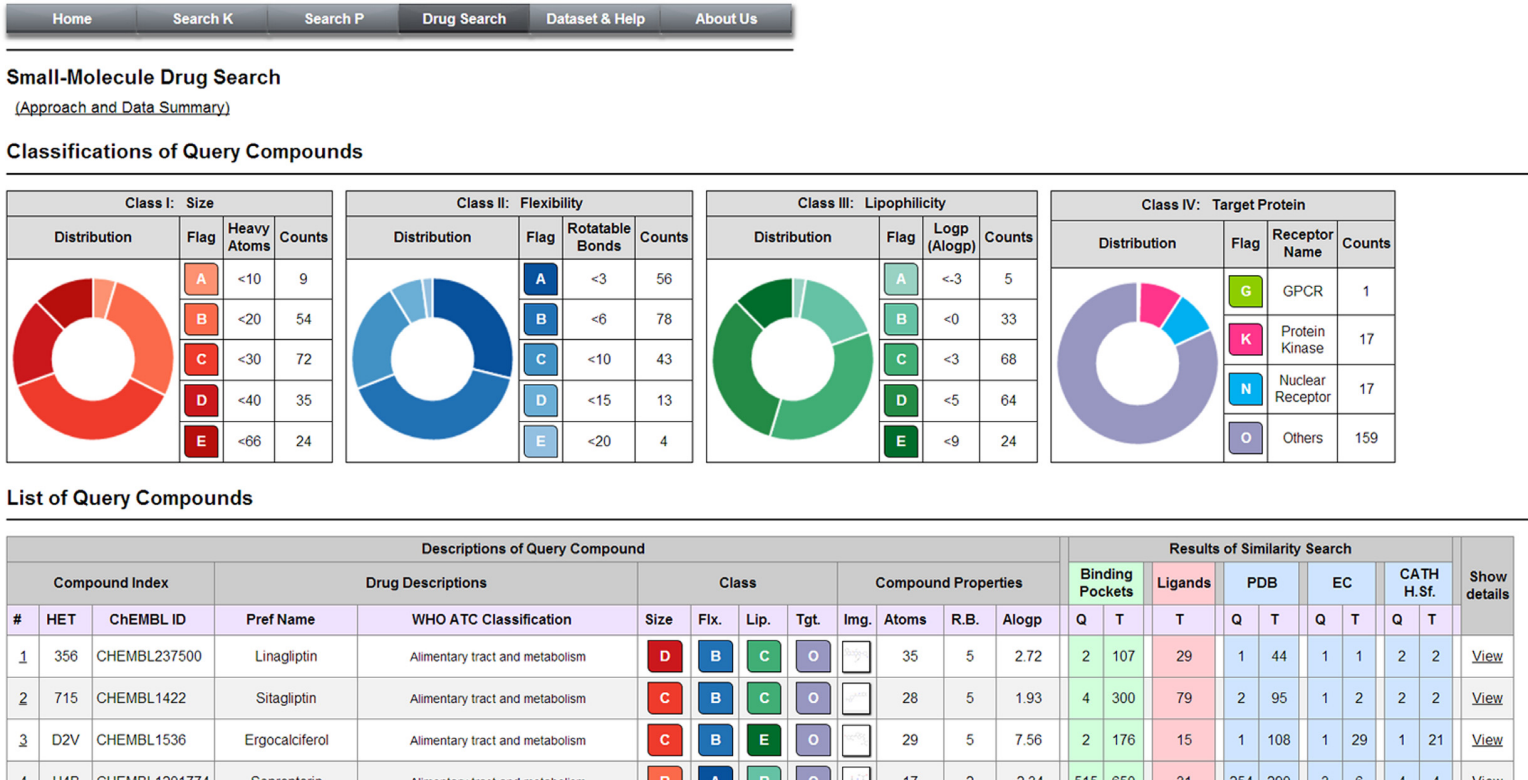
Start page of PoSSuMds. 194 approved drug compounds used as queries in this study are categorized in pie charts (Top) and listed in a table (Bottom).

The PoSSuMds interface starts with a table containing the 194 query drugs along with the drug descriptions and chemical properties (http://possum.cbrc.jp/PoSSuM/drug_search/) as presented in Figure 2. The query drugs have been categorized into five classes (A, B, C, D or E) according to their size, flexibility and lipophilicity, and can be sorted by the assigned class label. The drugs have also been categorized into four classes (G, K, N or O) based on their receptor types: GPCR (CATH: 1.20.1070.10), Protein Kinase (CATH: 1.10.510.10, 3.30.200.20), Nuclear Receptor (1.10.565.10) and ‘others’. Users can select a query drug of interest and then proceed to view the result of similarity searches.

Each result page is subdivided into four sections. The first section summarizes drug descriptions based on the WHO ATC classification and chemical properties of the query drug, and is followed by a table of statistics related to the results of the pocket similarity searches, including binding pockets, binding ligands and receptor proteins.

**Figure 3.**
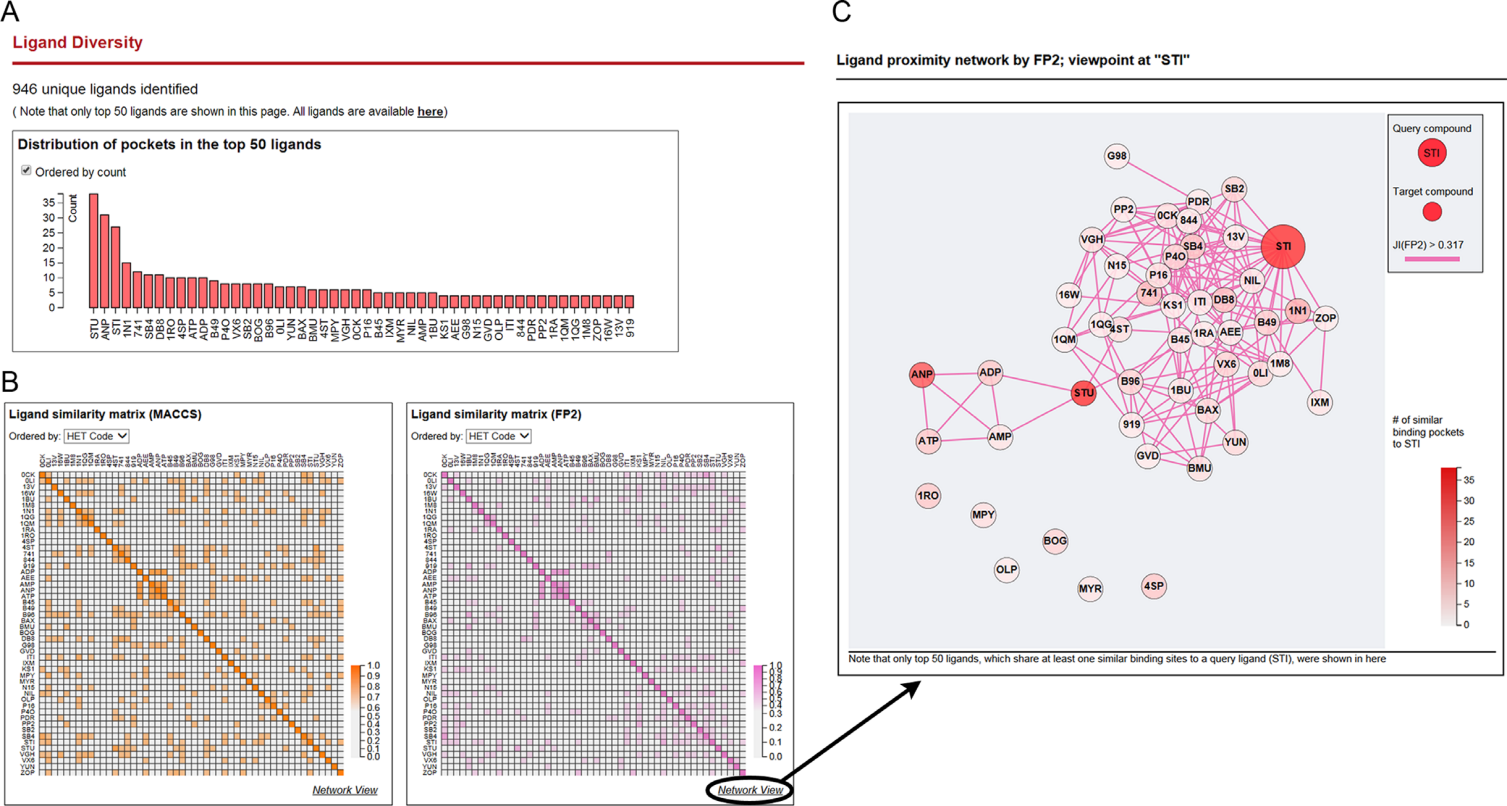
Captured images related to ligand diversity when the query compound was set to imatinib (HET: STI). The top 50 ligands, which are ranked by the number of binding pockets, are shown in the bar plot (**A**). Chemical similarities between the ligands are shown in Heatmaps (**B**) and in a Network view (**C**).

The variety of the retrieved ligands is shown in the second section. At the top of this section, the distribution of the retrieved ligands is displayed in a bar plot (Figure 3A). Chemical similarities among the ligands can be viewed as heatmaps (Figure 3B), where darker colors represent higher JI values. The relationships can also be visualized as a network (Figure 3C), where a ligand is denoted by a node and the chemical similarity is represented by an edge. This type of visualization is expected to be useful for understanding the distribution of ligand analogs that bind to structurally similar pockets. In the case of imatinib (HET: STI), an example of a typical kinase inhibitor, the chemical similarities to natural ligands, metabolites (e.g. ATP, ADP and AMP) and to other inhibitors such as dasatinib (HET: 1N1) and sunitinib (HET: B49), are apparent in the network view. Furthermore, up to 50 of the top ligands, in descending order of the number of binding pockets, are shown in the table at the end of this section. All the other ligands can also be downloaded at the end of this table.

The third section presents a description of the diversity of receptor proteins. The distributions of the target binding pockets in PDB entries and sequence groups (UniProt IDs and UniRef50 IDs) are summarized in bar plots (Supplementary Figure S2A), which are followed by pie charts representing the distributions of functional and structural groups (Supplementary Figure S2B). Unique groups such as EC codes observed only on the target side are shown in highlighted colors.

**Figure 4.**
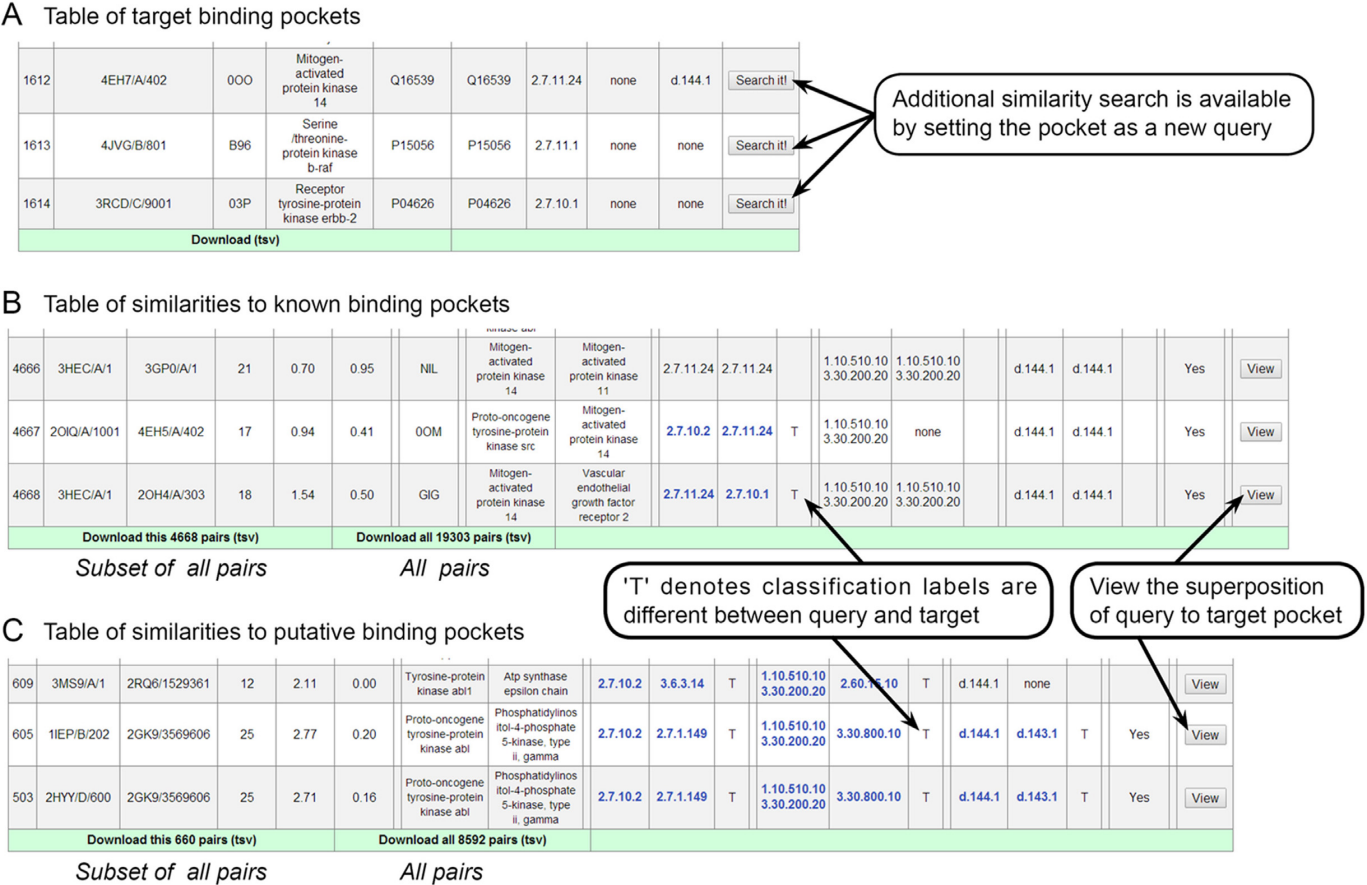
Table of binding pockets detected to be similar to the query pockets (**A**). Similar pocket pairs are displayed in two tables, depending on whether the similar pocket is a known binding pocket (**B**) or a putative pocket (**C**).

In the fourth and last section, one can retrieve all of the binding pockets on both the query and target sides (Figure 4A), as well as all of the similarity details between them (Figure 4B and C). For several query ligands, such as kinase inhibitors, the number of similar pocket pairs exceeds 10 000, which is difficult to display in a web page. Therefore, we generated a subset of pocket pairs in the following manner. Presuming that a query pocket is associated with UniProt ID P1 and HET code H1, and that one similar pocket was identified as associated with UniProt ID P2 and HET code H2, if multiple similar pocket pairs were identified between (P1, H1) and (P2, H2), then only the pair with the longest aligned length is selected. For putative pockets, the HET code H2 was ignored. If the interaction between a query ligand and an identified target receptor has been tested by any binding assay, then we assigned a flag ‘Yes’ to the similarity pair in the last column (Figure 4B and C). ChEMBL assay information was retrieved via the TargetMine data warehouse (22). Users can download not only the subset displayed in the tables, but also all pairs at the end of the tables (Figure 4B and C).

**Figure 5.**
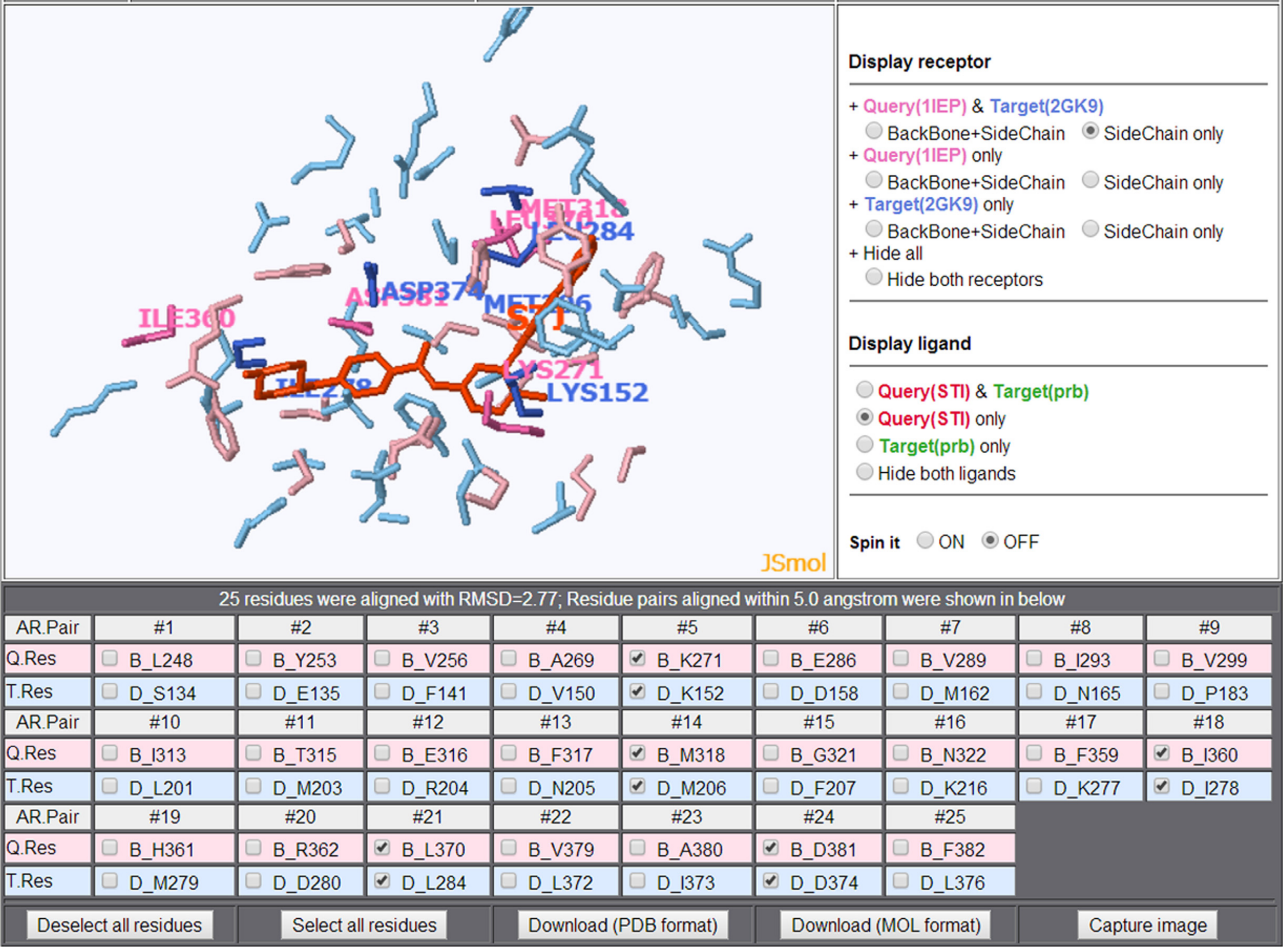
Captured image from the superimposition viewer, which shows the superimposition of an STI-binding pocket of 1IEP to a putative pocket of 2GK9. The query ligand and its pocket are shown in red and pink, and the target pocket is shown in cyan.

We present an example here: similarity between the imatinib (HET: STI)-binding pocket of the tyrosine protein kinase ABL (PDB ID: 1IEP) (23) and a putative pocket of phosphatidylinositol-4-phosphate 5 (P4P5)-kinase (PDB ID: 2GK9). Despite adopting different folds (with CATH codes ‘1.10.510.10, 3.30.200.20’ and ‘3.30.800.10’, respectively) (Figure 5 and Supplementary Figure S3), 25 residues of the two pockets are aligned with an Root-Mean-Square Deviation (RMSD) of 2.77 Å, suggesting that imatinib would bind to P4P5-kinase, which is consistent with its known Kd value of 380 nM (24).

Because PoSSuMds is fully integrated into PoSSuM, users can identify a target pocket similar to one of the binding pockets for the 194 compounds and proceed to additional searches and further investigation (Figure 4A).

## SUPERIMPOSITION VIEWER

In addition to browsing the statistical results, users can visualize individual superimposed pocket pairs (Figures 4B, C and 5). We have improved the superposition page, where the user can display/undisplay the query, the target, and can check corresponding amino acids based on the structural alignment, and can also download the 3D coordinates. As a demonstration, Figure 5 shows superimposed binding pockets for the example described in the section above. For the 3D molecular viewer, we employed JSmol (25), which was developed based on the HTML5 technology and which requires no enabling of Java in the user's web browser.

## DISCUSSION AND FUTURE DIRECTIONS

In this update, we specifically examined approved small-molecule drugs. We plan to expand the list of the query ligands to oral drugs, drug candidates and metabolites in the future. We currently use TM-align (26), which can align similar binding sites only in a sequence-order-dependent manner. However, fast and efficient pocket comparison methods have been proposed recently (27,28). Such methods should be used to compare poorly aligned pocket pairs by TM-align. Adopting such methods is expected to enhance our database. Another crucial factor is flexibility. Some drug compounds can bind to their receptors in various conformations, which in turn changes the shape and size of the binding pockets. To capture conformational changes, it is necessary to employ a method that incorporates binding pocket flexibility.

## SUPPLEMENTARY DATA

Supplementary Data are available at NAR Online.
